# Comprehensive genome-wide identification and expression analysis of the *EPF/EPFL* gene family in oat

**DOI:** 10.1186/s12864-025-11585-y

**Published:** 2025-06-02

**Authors:** Qingxue Jiang, Lin Ma, Siyuan Guo, Xinyue Zhou, Zhipeng Zhang, Yanhong Cui, Jun Tang, Dengxia Yi, Xuemin Wang

**Affiliations:** 1 Institute of Animal Sciences, Chinese Academy of Agricultural Sciences, Beijing, 100193 China; 2 Faculty of Science, McMaster University, Hamilton, L8S 4L8 Canada

**Keywords:** *Avena sativa*, *EPF/EPFL*, Drought stress, Salt stress, Expression analysis

## Abstract

Epidermal pattern factor-like (*EPF/EPFL*) genes are a unique class of small, secreted peptides found in plants that play crucial roles in plant stress responses. A genome-wide analysis revealed 33 *AsEPF/EPFL* genes in oats (*Avena sativa*), with 28 containing the conserved EPF domain and 5 harbouring the stomagen domain. These proteins share 2–6 conserved motifs, reflecting functional modularity. The phylogenetic classification grouped these genes into five evolutionarily conserved clades containing both monocot and dicot homologues, indicating early divergence prior to monocot–dicot speciation. Expression profiling revealed distinct tissue-specific patterns: preferential expression in roots (12 genes), stems (6 genes), leaves (5 genes), and spikes (7 genes), with 3 genes showing dual peak expression in stems and leaves. Further analysis of gene expression under salt and drought stress revealed that *AsEPF/EPFLs* are induced by both types of stress, with different genes showing varying expression patterns under drought and salt stress. This study identified valuable candidate genes for high-yielding and stress-resistant oat breeding.

## Background

Plants are immobile, which means they must endure abiotic stresses such as drought, salinity, and extreme temperatures. These stressors significantly limit their distribution and impact their growth and development [1]. Most of the water absorbed by plants is lost to the atmosphere through stomata, and regulating transpiration through stomatal control is an effective strategy to increase plant drought resistance [2]. Stomata play key roles in regulating carbon dioxide uptake and water loss [3]. In recent years, small peptides encoded by the epidermal patterning factor (EPF/EPFL) gene family have been identified as key factors in plant responses to abiotic stress [4]. Naoki et al. revealed, through modelling, that *EPF/EPFL* peptides share a common three-dimensional structure composed of a framework and a loop [5]. These peptides interact with signalling pathways associated with stomatal development to regulate stomatal density and opening/closing, thereby increasing plant adaptability under stress conditions.

Genetic and physiological analyses have shown that *EPF/EPFL* genes have great potential for improving plant WUE and drought tolerance by regulating stomatal development [6]. Recent studies have shown that regulating stomatal development through molecular methods can significantly increase plant drought resistance. Compared with the wild type strain, the Dunn team reported a dual effect after the *TaEPF1B* gene was overexpressed in wheat: the stomatal density decreased by 39%, whereas the water use efficiency increased by 27% [7]. Research on poplar has revealed a more complex regulatory mechanism. In the 84 K variety, the overexpression of PdEPFL6 not only reduced the stomatal density by 42% but also significantly increased leaf water retention by balancing the transpiration rate (decreased by 58%) and photosynthetic efficiency (maintained at 85% of normal levels) [8]. In potato, overexpression of the *StEPF2* gene led to unique phenotypic characteristics. Under drought stress, the stomatal density decreased by 35%, but the optimization of stomatal opening and closing rhythms successfully maintained the leaf water content at more than 90% of normal levels [9]. Notably, heterologous expression of the poplar *PdEPF3* gene in *Arabidopsis* revealed the feasibility of cross-species regulation. Transgenic plants presented a 22% increase in the photosynthetic rate and a 41% reduction in the transpiration rate under drought conditions, indicating excellent drought resistance phenotypes [10]. Li et al. found that hydrogen sulfide (H₂S) can modify *EPF2* and *EPFL9* through direct cysteinylation, affecting their protein activities and thereby influencing stomatal development in *Arabidopsis*, increasing the stomatal density in the lower epidermis of leaves [11]. These results indicate that the *EPF/EPFL* gene family plays an important role in regulating stomatal density, thereby affecting plant drought tolerance and water use efficiency. In addition, the *EPF/EPFL* gene family has been shown to participate in the plant inflorescence structure, leaf morphology, pollen tube elongation and other morphogenesis processes [12]. Sun et al. performed a qRT‒PCR analysis, which revealed that the expression of the *TaEPFL1* gene in the stamens (PS) of the wheat male sterile mutant hts-1 (exhibiting homeotic transformation of anthers into pistils) was significantly upregulated. Specifically, *TaEPFL1* levels in PS stamens were approximately 20-fold higher than those in normal pistils (P) and 15-fold higher than those in normal stamens (S). These results suggest that *TaEPFL1* overexpression may contribute to the homeotic transformation of stamens into pistils in wheat [13].

Given the crucial role of *EPF/EPFL* gene family members in regulating plant stress responses, members of the *EPF/EPFL* gene family have been systematically identified in various plants. HARA et al. first identified 11 *EPF/EPFL* gene family members in *Arabidopsis thaliana*, namely, *AtEPF1*, *AtEPF2*, *AtEPFL1*, *AtEPFL2*, *AtEPFL3*, *AtEPFL4*, *AtEPFL5*, *AtEPFL6*, *AtEPFL7*, *AtEPFL8*, and *AtEPFL9* [14]. *EPF/EPFL* gene family members have subsequently been identified in different plants, such as tomato (*Solanum lycopersicum*, 14 genes) [15], rye (*Secale cereale*, 12 genes) [16], barley (*Hordeum vulgare*, 11 genes) [17], rice (*Oryza sativa*, 12 genes) [18], maize (*Zea mays*, 17 genes) [18], and wheat (*Triticum aestivum*, 35 genes) [19]. While the complete oat genome was not fully sequenced and published until 2022 due to its large size (10.56 Gb) and complex hexaploid structure, the comprehensive genome-wide identification of *EPF/EPFL* family genes in *Avena sativa* remains to be completed [20].

Oat is an annual herbaceous plant of the Poaceae family that is widely grown as a dual-purpose crop for grain and fodder, particularly in cool temperate regions [21]. Oats possess excellent characteristics, such as tolerance to poor soils, salinity, cold, and drought; high forage yields; and high nutritional contents, making them valuable in livestock production [22]. Nevertheless, under the agricultural context of ensuring food security and maintaining a red line of 1.8 billion mu (120 million hectares) of arable land in China, oats are often cultivated in marginal lands prone to drought and salinity. Existing varieties struggle to meet the industrial demands. The *EPF/EPFL* gene family plays an important role in plant adaptation to adverse stress and growth and development processes. Therefore, identifying and exploring drought- and salt tolerance-related *EPF/EPFL* genes in oats is crucial for utilizing biotechnological approaches to breed new oat germplasms, supporting the development of drought-resistant and salt-tolerant oat varieties.

This study employed bioinformatics methods to systematically identify *EPF/EPFL* gene family members in the oat genome. The physicochemical properties, chromosomal localization, conserved domains, and gene structures of these family members were analysed. Furthermore, a clustering analysis was performed to elucidate the phylogenetic relationships of these family members in plants. Additionally, qRT‒PCR was employed to investigate the tissue-specific expression patterns of *AsEPF/EPFL* gene family members and their responses to drought and salt stress. These findings lay a foundation for a deeper understanding of the biological functions of the *EPF/EPFL* gene family in oats and provide important candidate genes for the discovery of key genes related to drought and salt tolerance in oats.

## Materials and methods

### Plant materials and methods

Seeds of the *Avena sativa* L. cultivar ‘Mengyan 1’ were selected for this study. Seeds were germinated in Petri dishes, transferred to seedling pots, and cultivated in a greenhouse with the relative humidity maintained between 65% and 75%. When the seedlings reached the three-leaf stage, they were subjected to drought and salt stress treatments. Drought stress was simulated using 20% PEG 6000, and samples were collected at 0 h, 4 h, 8 h, 12 h, and 24 h after treatment. Salt stress was induced by adding 150 mM NaCl, and samples were collected at 0 h, 3 h, 6 h, 12 h, and 24 h after treatment. Additionally, tissues (roots, stems, leaves, and spikes) from oats at the heading stage were sampled. All the samples were immediately frozen in liquid nitrogen and stored at -80 °C for subsequent RNA extraction. Each sample had three biological replicates.

### Identification and analysis of the *EPF/EPFL* gene family

The protein sequences of 11 *AtEPF/EPFL* genes were downloaded from the *Arabidopsis* genome database TAIR (https://www.arabidopsis.org/). Using TBtools v1.121 software, BLASTP searches were performed against the oat genome to identify AsEPF/EPFL proteins (E value < 1e^− 5^). Whole-genome data, CDSs, protein sequences, and annotation files for oats were downloaded from the Ensembl Plants database (https://plants.ensembl.org/). The Hmm search tool in TBtools v1.121 was used to further search for and identify oat EPF/EPFL proteins. The protein sequences obtained via both methods were merged, redundant sequences were removed, and the final set of oat *EPF/EPFL* gene family members was obtained. The Pfam (http://pfam.xfam.org/) and NCBI-CDD (https://www.ncbi.nlm.nih.gov/cdd) websites were used for additional protein identification. TBtools v1.121 software was used to predict physicochemical properties such as the molecular weight and isoelectric point of the AsEPF/EPFL protein sequences.

### Conserved motif and gene structure analyses

TBtools v1.121 was used to analyse the gene structures of *AsEPF/EPFL* family members. MEME online software (http://meme.nbcr.net/meme) was used to analyse conserved motifs in AsEPF/EPFL proteins, with the maximum motif count set to 7. The results were visualized using TBtools v1.121.

### Chromosomal localization analysis of the *EPF/EPFL* gene family

Chromosome density files were extracted using TBtools v1.121 Chromosome Heatmap Data. The locations of *AsEPF/EPFL* family genes on chromosomes were determined using the oat genome database, and their distribution on chromosomes was visualized using TBtools v1.121 Gene Location Visualize from GTF/GFF.

### Evolutionary analysis of the *AsEPF/EPFL* system

The protein sequences of 56 *EPF/EPFL* genes from oats, rice, *Arabidopsis* and *Physcomitrella patens* were aligned using MUSCLE in MEGA-X software. A neighbour-joining (NJ) phylogenetic tree was constructed using MEGA-X, and the resulting tree was visualized and beautified using the iTOL website (https://itol.embl.de).

### Synteny and homologous gene pairs

The collinearity of *EPF/EPFL* genes in *A. thaliana*, *T. aestivum*, and *A. sativa* was analysed using the One Step MCScanX module in TBtools v1.121. The Dual Synteny Plot plugin in TBtools was employed to visualize the collinearity of the *AsEPF/EPFL* genes. This visualization revealed the chromosomal distribution of *AsEPF/EPFL* genes, their collinear relationships, and their correspondence with *EPF/EPFL* genes in other species.

### Expression pattern of the EPF/EPFL genes in oat

Total RNA was extracted from different tissues and from samples subjected to drought and salt stress treatments using the Fast Pure Plant Total RNA Isolation Kit (TIANGEN, Beijing, China). cDNA was synthesized using the Trans Script II One-Step gDNA Removal and cDNA Synthesis Super Mix Kit (Vazyme, Nanjing, China). qRT‒PCR was performed using Hieff^®^ qPCR SYBR Green Master Mix (Yeasen, Shanghai, China) in a 20 µL reaction volume, which included 10.0 µL of SYBR Green Master Mix, 0.4 µL each of forwards and reverse primers, 8.2 µL of sterile water, and 1 µL of cDNA template. The PCR program included initial denaturation at 95 °C for 3 min, followed by 40 cycles of 95 °C for 15 s, 60 °C for 30 s, and 72 °C for 20 s. A melting curve analysis was performed from 60 °C to 95 °C. AsActin was used as the reference gene, and relative expression levels were calculated using the 2^−ΔΔCt^ method. All the experiments were performed with three biological replicates. The sequences of the primers used for qRT‒PCR are listed in Table 1.

This methodology section outlines the detailed procedures used for the identification, characterization, and expression analysis of the *AsEPF/EPFL* gene family in oats under various conditions.

### Data analysis

The qRT‒PCR data were processed using Excel 2019. GraphPad Prism software was used to generate heatmaps depicting the relative expression levels of the 33 *AsEPF/EPFL* genes across different tissues and under various abiotic stress conditions. Additionally, a clustering analysis was performed to analyse the expression patterns.

**Table 1:** qRT‒PCR primers for the *AsEPF/EPFL* genes

Primer name	Forward primer (5′-3′)	Reverse primer (5′-3′)
4AG0623540	CGCATGATGGAGGAAGGGAAG	AGTGTTGTTGTCAAGGGAATTTC
3DG0522310	CTCTTCTTCCTGTCGCATCTG	CCCTCCTCGTCTCATCCAG
7AG1219190	GCTGCCGTTGTTGTGCTATCTC	GCTCCGCTCCGTCATCGC
4AG0600120	GCGGAAGAAGAAGAAGGAGGAG	ACGGCTTGTAGTTGGAGTGG
4DG0743870	CGCCGAGGAGAAGAGCATTG	TAGTTGGAGTGGTCGTCGTAG
3AG0421080	CTCTTCTTCCTGTCGCACCTG	ATCCTCGGCACGCACCAG
6CG1093320	CCTGTCCACTTTTCAGAACCTATC	CACCCGAGCGGCTTGTAG
7DG1384010	GACTAGCAGCCGCACTACTTG	CGCACCTTCTCCTCCTCTCC
3DG0552300	GTGCTGACGGTGGTGGTG	CAAGGTGCTGGTGTTCGCC
2DG0337700	TCTTCCTCTCCTTGCTCCTCTTC	TCCTCCTCCTGCTCCTTATTGG
3CG0493720	GCTTGGCTCTTCTTCCTATCGC	ATCCTCGGCACGCACCAGG
7AG1224340	GACTAGCAGCCGCACTACTT	CGCACCTTCTCCTCCTCTC
1DG0129120	CGCTCCTCCTCCTCCTCTTC	AGCCTGTCGCCGCACTTG
1DG0167100	CGTCGTCGCCTCCCTCTG	CGCTGCTCCTGACATCTTCC
6DG1176190	CCCTCTCCACTTTTCAGAACC	CGAGCGGCTTGTAGTTGG
4DG0783280	TGCTCCTCTTCTTCCTCCTCTC	GGGTGGTTTCTCCTCGTTCAG
4DG0729490	GCTTCTCCTCTTCTCCTTCCTG	TCCTCCTCCTCGGCAATGG
1AG0016280	CCTTCCATCCATCCATCCACAG	GACGACGAGCAGCCTTAGC
2CG0320600	CGCAACACCTCATAGACATAGC	GAACACAGCCACGAGAAGAAG
7CG0695670	CTGCGGAAGAAGAAGAAGAAGAA	GTGGTCGTCGTAGGAAGAAGG
4CG1276840	CGCTCATCCTCCTCTTCCTC	CAGCCTGTCGCCGCACTTG
2AG0242220	GGAGGCGGCGGAGATGAG	GGCGATGGAGCACTTGAAGC
4DG0748330	AACTCCTGCTACTTCACTCCTC	ATCACCACCGCCGACATC
7CG0700440	CTGCTCCTCCTTCTGCTACC	ATCATCACCGCCGACACC
1AG0048570	GCGTCGTCGCCTCCCTCTG	GCTGCTCCTGACATCTTCC
7DG1389220	CTGCCGTTGTTGTGCTATCTC	GCTCCGCTCCGTCATCGC
6AG1023720	AGAACCTGGCGGAGGACAAG	GAGCGTCGTGGCAACCTG
4AG0632020	GTGCTGACGGTGGTGGTGG	CGCAAGGTGCTGGTGGTC
4AG0643770	GCTCCTCTTCTTCCTCCTCTCC	CTATATGTTCTCCTGTTAATCCTTGG
3CG0505940	CTACAGTGGCGAGTGAGACC	AGCGGCAGCAACAGTATGG
2CG0291330	TCTTCCTCTCCTTGCTCCTCTT	CCTCCTCCTGCTCCTTACTG
4DG0771310	GCTCCTCTTCTTCCTCCTCTC	TATATGTTCTCCTGTTAATCCTTGG
2AG0212650	CTTCCTCTCCTTGCTCCTCTTC	CCTCCTCCTGCTCCTTACTGG
AsActin	CACTGCCGAGCGGGAAATTG	TGATGGAAGGCTGGAAGAGGAC

## Results

### Identification and analysis of the physicochemical properties of the *EPF/EPFL* gene family

In the oat genome, a total of 33 nonredundant *EPF/EPFL* gene family members were identified (Table 2). The analysis of the physicochemical properties analysis the following characteristics of the AsEPF/EPFL proteins: (1) the length of the AsEPF/EPFL proteins ranged from 106 to 213 amino acids; (2) the molecular weights varied between 11,418.25 and 32,984.15 Da, with an average of 15,386.06 Da; (3) the isoelectric points (pI) ranged from 7.09 to 10.82; (4) the instability index of the AsEPF/EPFL proteins ranged from 44.8 to 87.54, indicating their stability; and (5) the aliphatic index ranged from 59.65 to 89.67. The hydrophilicity index ranged from − 0.447 to 0.02, with the average suggesting hydrophilic properties. These results indicate that AsEPF/EPFL proteins are predominantly composed of basic amino acids and exhibit hydrophilic characteristics (Table 2).

**Table 2:** Physical and chemical properties of AsEPF/EPFLs

ID	Chr Location	Amino Acids	Molecular Weight	Theoretical pI	Instability Index	Aliphatic Index	Grand Average of Hydropathicity
AVESA.00010b.r1.4AG0623540	4 A:341,292,372,341,293,956	312	32984.5	10.82	80.43	69.33	-0.23
AVESA.00010b.r1.3DG0522310	3D:26,890,21126,891,783	119	13208.2	10.03	67.06	80.25	-0.099
AVESA.00010b.r1.7AG1219190	7 A:138,739,243–138,740,181	120	13231.27	8.79	73.74	72.5	-0.236
AVESA.00010b.r1.4AG0600120	4 A:265,140,156–265,141,073	111	12086.13	9.69	62.5	79.28	-0.232
AVESA.00010b.r1.4DG0743870	4D:253,105,873–253,106,689	110	11928.96	9.69	60.9	80.91	-0.174
AVESA.00010b.r1.3AG0421080	3 A:69,461,343–69,461,808	119	13111.37	10.15	64.28	77.82	-0.206
AVESA.00010b.r1.6CG1093320	6 C:402,160,455–402,161,586	115	12368.17	9.46	53.31	62.17	-0.187
AVESA.00010b.r1.7DG1384010	7D:84,111,192–84,111,681	128	13356.33	8.6	61.74	79.38	-0.091
AVESA.00010b.r1.3DG0552300	3D:357,151,732–357,152,978	135	15116.65	10.05	87.54	69.93	-0.253
AVESA.00010b.r1.2DG0337700	2D:445,938,958–445,940,370	150	16521.38	8.75	50.88	89.67	0.007
AVESA.00010b.r1.7AG1224340	7 A:173,108,213–173,108,699	131	13509.49	8.85	60.74	80.53	-0.062
AVESA.00010b.r1.3CG0493720	3 C:441,104,824–441,106,014	119	13148.37	10.4	78.21	67.98	-0.316
AVESA.00010b.r1.1DG0129120	1D:442,739,192–442,740,419	106	11476.37	9.79	75.34	77.55	-0.076
AVESA.00010b.r1.6DG1176190	6D:131,330,530–131,331,426	115	12259.98	9.61	54.08	59.65	-0.276
AVESA.00010b.r1.1DG0167100	1D:307,877,586–307,878,947	131	13518.62	9.25	63.9	68.47	-0.087
AVESA.00010b.r1.4DG072949	4D:177,100,140–177,101,128	141	15669.04	9.46	67.78	78.94	-0.377
AVESA.00010b.r1.4DG0783280	4D:375,659,485–375,660,844	124	13680.55	7.09	55	70.89	-0.346
AVESA.00010b.r1.1AG0016280	1 A:454,487,915–454,490,395	212	22026.48	9.71	55.93	75.47	-0.005
AVESA.00010b.r1.7CG0695670	7 C:77,733,004–77,733,897	116	12753.09	10.2	58.26	76.72	-0.398
AVESA.00010b.r1.2CG0320600	2 C:508,222,649–508,223,607	182	19158.44	8.93	58.28	84.34	0.232
AVESA.00010b.r1.4CG1276840	4 C:98,248,152–98,248,573	106	11418.25	9.79	81.42	71.13	-0.146
AVESA.00010b.r1.2AG0242220	2 A:387,377,357–387,378,429	131	13949.37	8.8	49.46	76.79	0.007
AVESA.00010b.r1.4DG0748330	4D:268,883,169–268,883,741	136	14330.34	8.9	46.33	76.84	-0.222
AVESA.00010b.r1.7CG0700440	7 C:54,446,612–54,448,524	126	13596.64	9.72	44.8	79.84	-0.245
AVESA.00010b.r1.1AG0048570	1 A:322,903,882–322,905,227	134	13877.98	9.1	72.51	63.96	-0.151
AVESA.00010b.r1.7DG1389220	7D:52,814,691–52,815,630	119	13200.29	8.78	71	77.98	-0.15
AVESA.00010b.r1.6AG1023720	6 A:172,085,798–172,087,124	117	12432.22	9.61	54.38	60.34	-0.218
AVESA.00010b.r1.4AG0632020	4 A:369,998,489–369,999,651	130	14531.99	9.78	88.54	66.62	-0.231
AVESA.00010b.r1.4AG0643770	4 A:420,590,561–420,591,913	191	20834.68	8.7	56.66	64.97	-0.406
AVESA.00010b.r1.3CG050594	3 C:526,163,238–526,164,924	227	24469.27	9.7	69.62	76.48	-0.152
AVESA.00010b.r1.2CG0291330	2 C:336,260,669–336,262,010	149	16356.12	8.52	51.96	83.09	-0.073
AVESA.00010b.r1.4DG0771310	4D:334,947,335–334,948,723	192	21118.01	8.83	60.38	64.11	-0.447
AVESA.00010b.r1.2AG0212650	2 A:242,310,696–242,312,142	150	16511.34	8.75	55.52	89	0.02

### Analysis of the conserved motifs and structures of the EPF/EPFL gene family

**Fig. 1:**
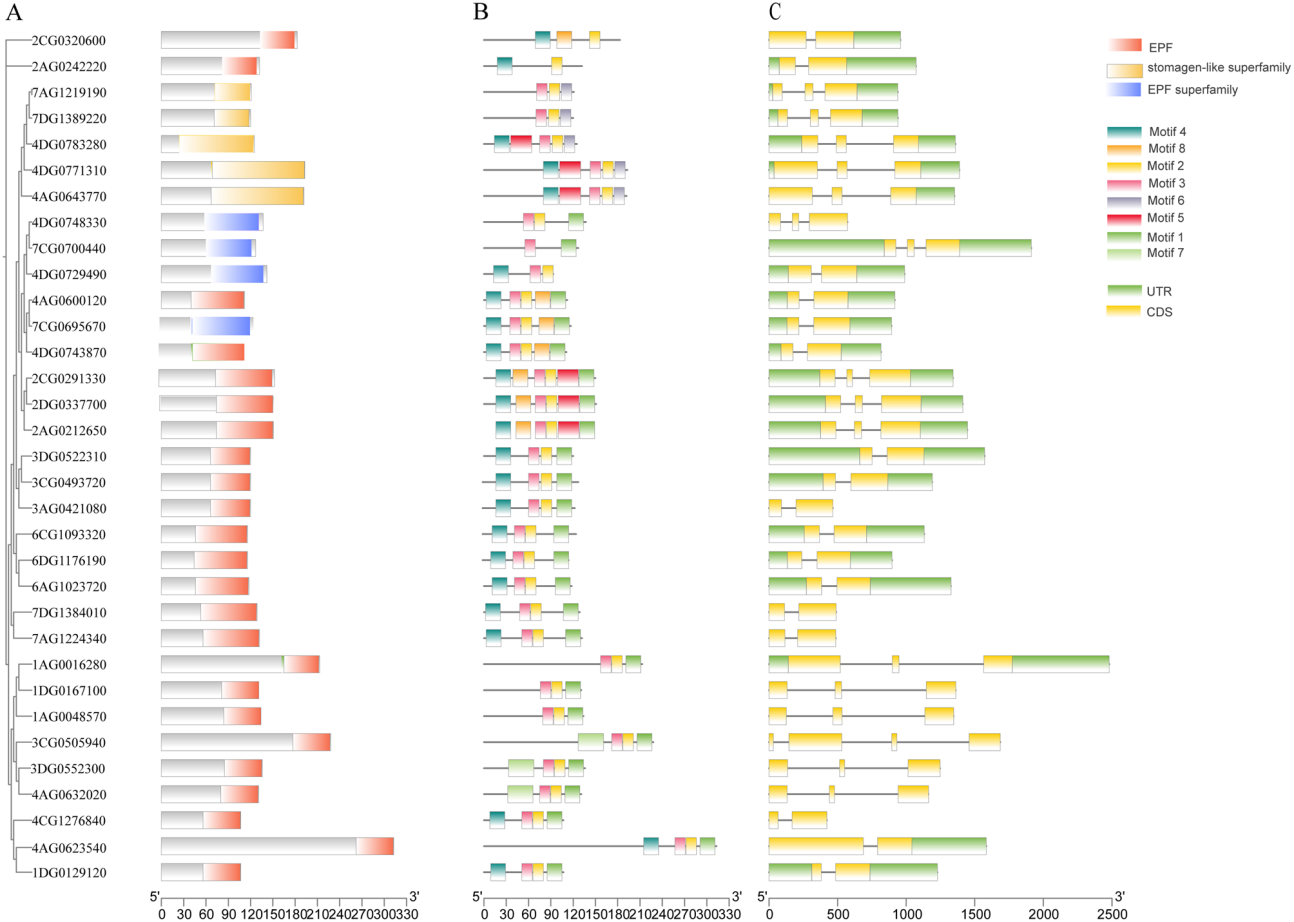
**A** Evolutionary tree and conserved domains of AsEPF/EPFL proteins. **B**. Conserved motifs of the *EPF/EPFL* gene family. **C**. Gene structure of the *EPF/EPFL* gene family

The results of the conserved domain analysis are shown in Fig. 1A. The conserved domain of 24 AsEPF/EPFL members is EPF; the conserved domain of 4 AsEPF/EPFL members (4DG0748330, 7CG0700440, 4DG0729490, and 7CG0695670) is stomagen; and the conserved domain of 5 AsEPF/EPFL members (7AG1219190, 7DG1389220, 4DG0783280, 4DG0771310, and 4AG0643770) is the stomagen-like superfamily. Further analysis of the conserved motifs of the AsEPF/EPFL proteins revealed 7 conserved motifs, named Motif 1 to Motif 7 (Fig. 1B). Among these, 17 AsEPF/EPFLs contain 4 motifs; 5 AsEPF/EPFLs contain 3 motifs; 2AG0242220 contains only 2 motifs, namely, Motif 4 and Motif 8; and 7CG0700440 contains only 2 motifs, namely, Motif 3 and Motif 1. In addition, by comparing the genomic sequences of *AsEPF/EPFL* genes with their corresponding CDSs, the structures of *AsEPF/EPFL* genes was obtained (Fig. 1C). Most (31) *AsEPF/EPFL* genes contain 2 exons and 1 intron, whereas *2CG0320600* and *4AG0643770* have only one exon. These results indicate that the *AsEPF/EPFL* gene family in oats is highly conserved.

### Chromosomal localization of the *EPF/EPFL* gene family

**Fig. 2:**
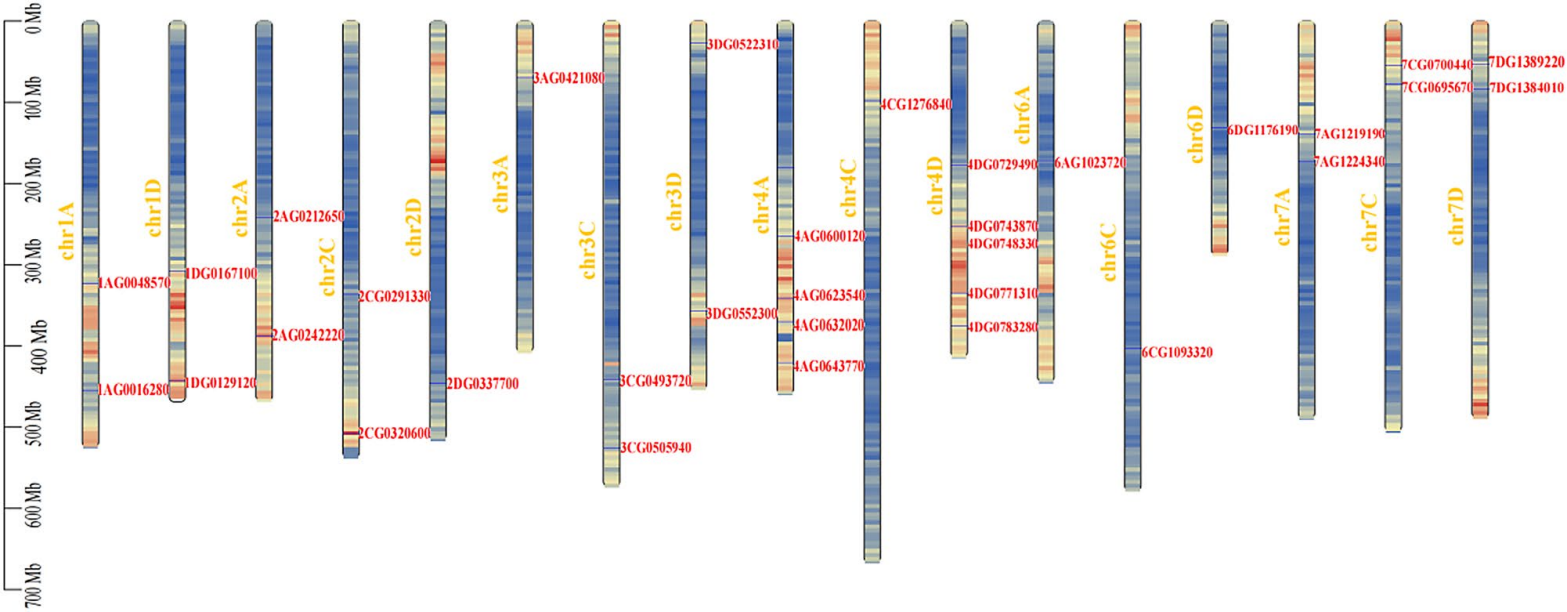
Chromosomal locations of *AsEPF/EPFL* genes.Gene density is depicted in a color gradient, with red indicating regions of higher gene density and blue indicating regions of lower gene density

The chromosomal localization analysis revealed that the 33 *AsEPF/EPFL* genes are located on 17 chromosomes of the oat genome (Fig. 2). Further analysis indicated that these genes are unevenly distributed across the 17 chromosomes. Chromosome 4D contains 5 *AsEPF/EPFL* genes, namely, *4DG0743870*, *4DG0783280*, *4DG0729490*, *4DG0748330*, and *4DG0771310*; chromosome 4 A contains 4 *AsEPF/EPFL* genes, namely, *4AG0623540*, *4AG0600120*, *4AG0632020*, and *4AG0643770*; chromosomes 1 A, 1D, 2 A, 2 C, 3 C, 3D, 7 A, 7 C, and 7D each contain 2 *AsEPF/EPFL* genes; and chromosomes 2D, 3 A, 4 C, 6 A, 6 C, and 6D each contain only 1 *AsEPF/EPFL* gene. These results indicate that no gene duplication occurred during the formation of the *AsEPF/EPFL* genes in the oat genome.

### Phylogenetic analysis of the *EPF/EPFL* gene family

**Fig. 3:**
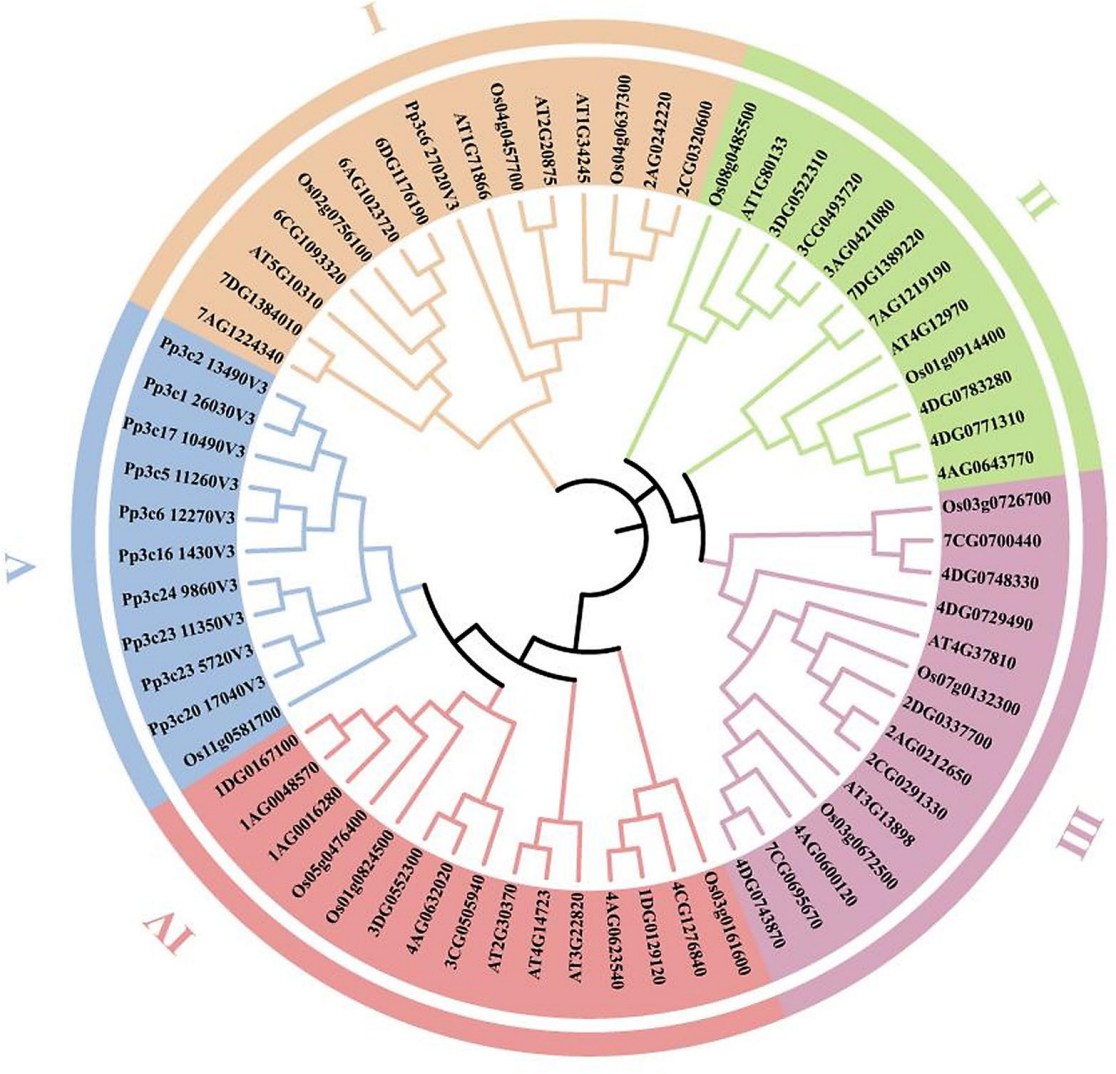
Phylogenetic tree of *EPF/EPFL* genes.Clades I–IV are color-coded to indicate the evolutionary groupings of the EPF/EPFL proteins. *Note*: The accession numbers for the *OsEPF1-12* genes of the rice *EPF/EPFL* family are as follows: LOC_Os03g0161600, LOC_Os11g0581700, LOC_Os05g0476400, LOC_Os01g0824500, LOC_Os04g0457700, LOC_Os04g0637300, LOC_Os08g0485500, LOC_Os02g0756100, LOC_Os01g0914400, LOC_Os03g0726700, LOC_Os03g0672500, and LOC_Os07g0132300. The accession numbers for the *AtEPF1-2* genes of the *Arabidopsis EPF/EPFL* family are AT2G2075 and AT1G34245. The accession numbers for the *AtEPFL1-9* genes are AT5G10310, AT4G37810, AT3G13898, AT4G14723, AT3G22820, AT2G30370, AT1G71866, AT1G80133, and AT4G12970. The accession numbers for the *PpEPF* genes are Pp3c24_9860V3, Pp3c23_11350V3, Pp3c23_5720V3, Pp3c20_17040V3, Pp3c17_10490V3, Pp3c5_11260V3, Pp3c16_1430V3, Pp3c1_26030V3, Pp3c2_13490V3, Pp3c6_27020V3, and Pp3c6_12270V3

Multiple sequence alignments were performed on the amino acid sequences of the 33 AsEPF/EPFL proteins from oats, 12 OsEPF/EPFL proteins from rice, 11 *PpEPF/EPFL proteins* from moss plants (*Physcomitrella patens*) and 11 AtEPF/EPFL proteins from *Arabidopsis*, totalling 67 EPF/EPFL proteins, to explore the evolutionary relationships of the *EPF/EPFL* gene family members in oats and other species. A phylogenetic tree was constructed (Fig. 3). The results of the phylogenetic analysis presented in Fig. 3 show that the *EPF/EPFL* family can be classified into five distinct subgroups. Subgroups II, III, and IV contain conserved *EPF/EPFL* members from *Arabidopsis* (eudicot), rice (*Oryza sativa*; monocot), and oats (*Avena sativa*; monocot), with *AsEPF/EPFL* sequences exhibiting closer phylogenetic relationships to the *OsEPF/EPFL* sequences within each subgroup. Notably, *P. patens* is exclusively clustered in subgroups I and V.

### Collinearity analysis of *EPF/EPFLs* in oat, *arabidopsis*, and wheat

**Fig. 4:**
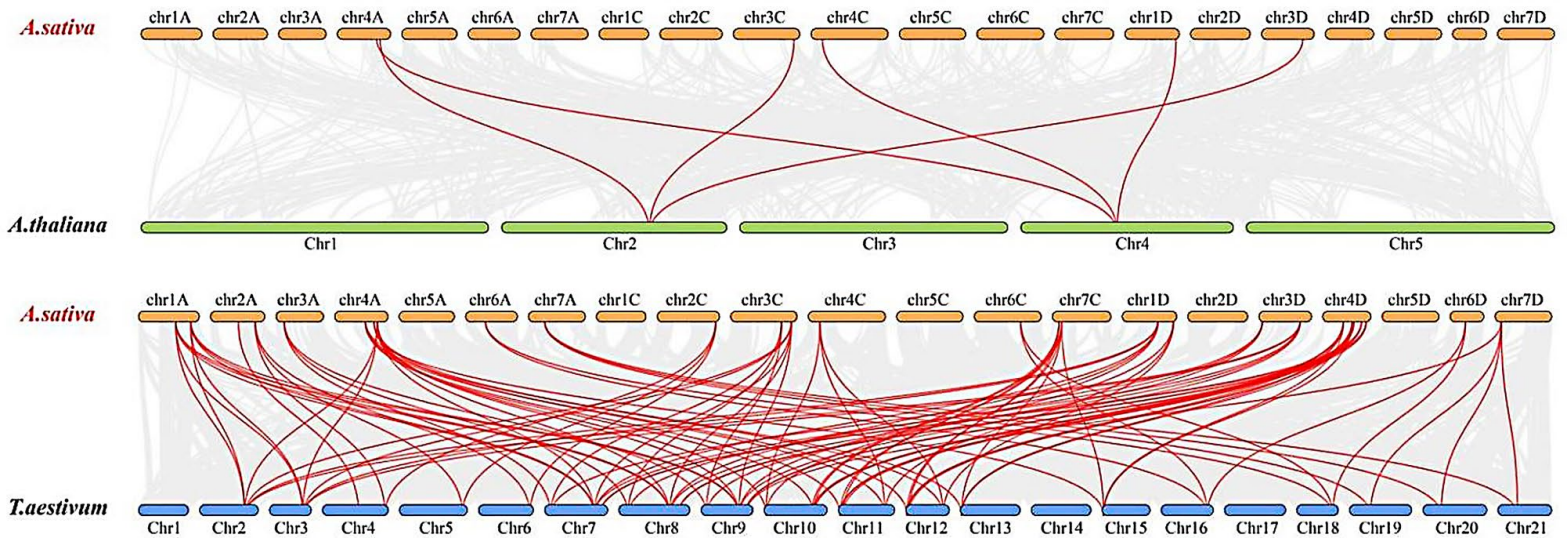
Covariance analysis of *EPF/EPFL* genes from *A. sativa*, *A. thaliana* and *T.aestivum.* The grey lines in the background indicate blocks of collinearity within *A. sativa* and the indicated plants, whereas the red lines indicate homozygous *EPF/EPFL* gene pairs

We further investigated the evolutionary history of the *EPF/EPFL* gene family across different species by conducting a comprehensive collinearity analysis of the genomes of *A. sativa*, *A. thaliana*, and *T. aestivum*. The results of the analysis, as shown in Fig. 4, revealed significant gene collinearity among these three species. Specifically, six pairs of collinear genes were identified between oat and *A. thaliana*: *4AG0632020*, *4AG0623540*, *3CG0505940*, *4CG1276840*, *1DG0129120*, and *3DG0552300*. These genes are unevenly distributed across chromosomes 4 A, 3 C, 4 C, 1D, and 3D in oats and other chromosomes.

In contrast, the collinearity between oat and wheat was much stronger, with 100 pairs of collinear genes identified. This finding suggests a closer evolutionary relationship of the *EPF/EPFL* gene family between oat and wheat than in *A. thaliana*. Further examination revealed that the *EPF/EPFL* genes in the oat genome are widely distributed across 16 of its chromosomes, whereas no *EPF/EPFL* genes were detected on the other five chromosomes (5 A, 1 C, 5 C, 2D, and 5D).

### Expression patterns of *EPF/EPFL* genes in different oat tissues

**Fig. 5:**
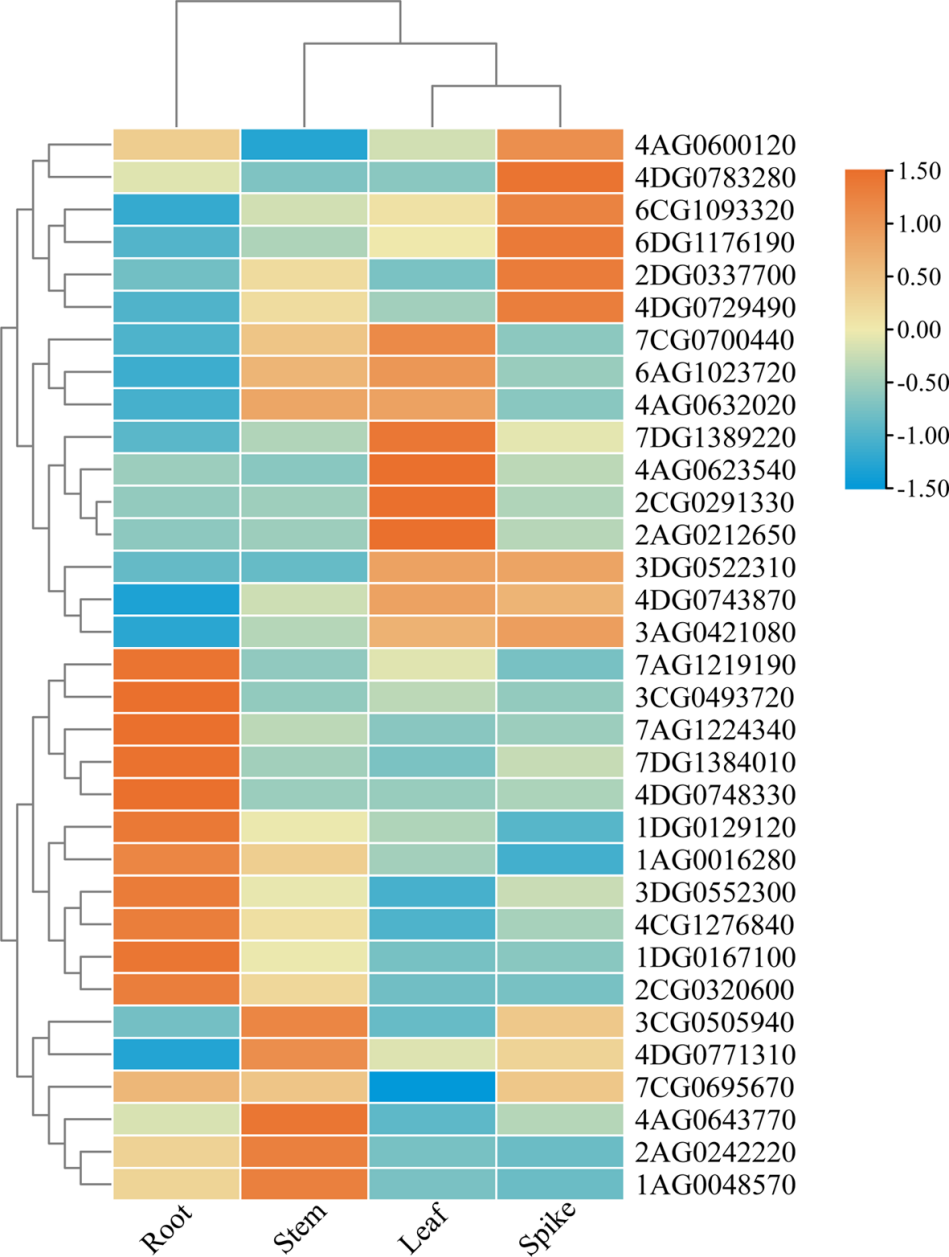
Expression patterns of *AsEPF/EPFL* genes in different oat tissues

The expression patterns of 33 *AsEPF/EPFL* genes in different oat tissues (roots, stems, leaves, and spikes) were analysed using qRT‒PCR. As shown in Fig. 4, the expression levels of the 33 *AsEPF/EPFL* genes varied significantly across the four tissues. Twelve genes (*7AG1219190*, *3CG0493720*, *7AG1224340*, *7DG1384010*, *4DG0748330*, *1DG0129120*, *1AG0016280*, *3DG0552300*, *4CG1276840*, *1DG0167100*, *2CG0320600*, and *7CG0695670*) presented the highest expression levels in roots; six genes (*4AG0632020*, *3CG0505940*, *4DG0771310*, *4AG0643770*, *2AG0242220*, and *1AG0048570*) presented the highest expression levels in stems; five genes (*7CG0700440*, *7DG1389220*, *4AG0623540*, *2CG0291330*, and *2AG0212650*) presented the highest expression in leaves; and seven genes (*4AG0600120*, *4DG0783280*, *6CG1093320*, *6DG1176190*, *4DG0729490*, *2DG0337700*, and *3AG0421080*) presented significantly higher expression levels in spikes than in other tissues. Additionally, *7CG0700440*, *6AG1023720*, and *4AG0632020* presented significantly higher expression in stems and leaves than in roots and spikes(Fig. 5). The differing expression levels across tissues suggest that the 33 *AsEPF/EPFL* genes may perform distinct biological functions in various oat tissues.

### Characteristics of *EPF/EPFL* expression in response to abiotic stress

**Fig. 6:**
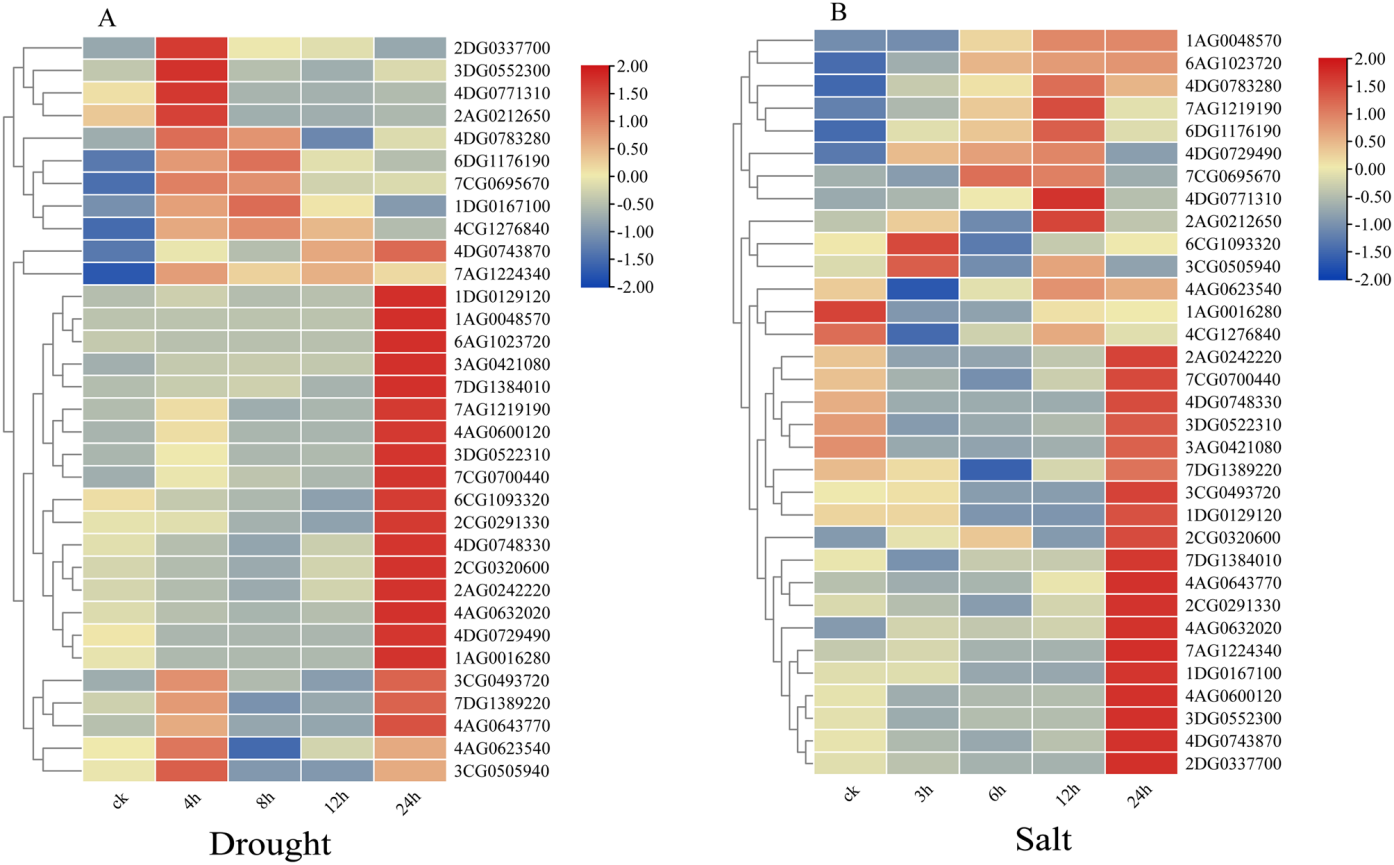
(**A**) Relative expression of the *AsEPF/EPFL* genes under PEG stress. (**B**) Relative expression of the *AsEPF/EPFL* genes under NaCl stress.ck: non-stressed control plants harvested at matching time points

The expression patterns of 33 *AsEPF/EPFL* genes under drought stress were analysed using qRT‒PCR. As shown in Fig. 5A, all 33 *AsEPF/EPFL* genes presented varying degrees of upregulation under drought stress (Fig. 6A). Among them, 9 *AsEPF/EPFL* genes (*2DG0337700*, *3DG0552300*, *4DG0771310*, *2AG0212650*, *4DG0783280*, *7CG0695670*, *7AG1224340*, *4AG0623540*, and *3CG0505940*) peaked at 4 h poststress; *6DG1176190* and *1DG0167100* peaked at 8 h; and 22 *AsEPF/EPFL* genes (*4DG0743870*, *1DG0129120*, *1AG0048570*, *6AG1023720*, *3AG0421080*, *7DG1384010*, *7AG1219190*, *4AG0600120*, *3DG0522310*, *7CG0700440*, *6CG1093320*, *2CG0291330*, *2CG0320600*, *2AG0242220*, *2CG0320600*, *4DG0748330*, *4AG0632020*, *4DG0729490*, *1AG0016280*, *3CG0493720*, *7DG1389220*, and *4AG0643770*) peaked at 24 h. These results suggest that *AsEPF/EPFL* genes may participate in the oat drought stress response through different regulatory pathways.

In response to salt stress, the qRT‒PCR analysis revealed that only 2 *AsEPF/EPFL* genes (*1AG0016280* and *4CG1276840*) were downregulated, whereas the remaining 31 *AsEPF/EPFL* genes were upregulated to varying degrees (Fig. 6B). Among these, *6CG1093320* and *3CG0505940* peaked at 3 h poststress; *7CG0695670* peaked at 6 h; 8 *AsEPF/EPFL* genes (*1AG0048570*, *4DG0783280*, *7AG1219190*, *6DG1176190*, *4DG0729490*, *4DG0771310*, *2AG0212650*, and *4AG0623540*) peaked at 12 h; and the remaining 20 *AsEPF/EPFL* genes peaked at 24 h (Fig. 6B). These results indicate significant differences in the response times of different *AsEPF/EPFL* genes to salt stress.

## Discussion

Plant epidermal patterning factor-like (*EPF/EPFL*) peptides are small secretory peptides that are widely present in plants and play crucial roles in growth, development, and stress resistance [23]. Previous studies have shown that some signal peptides are involved in signalling during plant growth and development, whereas others participate in defence responses or symbiotic processes [24, 25]. In this study, we utilized bioinformatics tools to identify 33 *AsEPF/EPFL* genes in the hexaploid oat genome. Research has revealed significant differences in the number of family members across species, ranging from diploid species (*A. thaliana* with 11 genes, *H. vulgare* with 11 genes, and *Z. mays* with 17 genes) to polyploid species (*T. aestivum* with 35 genes and *O. sativa* with 33 genes). The relatively high number of *AsEPF/EPFL* genes in oat may be attributed to its complex polyploid evolutionary history (2n = 6x = 42, AADDCC), with its genome originating from interspecific hybridization between a tetraploid (CCDD) and a diploid (AA) ancestor [26]. The phylogenetic analysis revealed that *AsEPF/EPFLs* can be divided into five subgroups. Notably, *P. patens* was found only in branches I and V, indicating that these two branches may have originated after the evolution of *pteridophytes.* Although lower bryophytes and ferns have not yet developed stomata and vascular tissues, members of the *EPF/EPFL* gene family have already emerged. The increase in the number of *EPF/EPFL* gene family members throughout evolution is likely attributable to gene or genome duplication. Overall, the phylogenetic analysis suggested that the *EPF/EPFL* family originated before the divergence of monocots and dicots, with the biological functions of orthologous genes being conserved across different species.

Although there are few reports on *EPF/EPFL* genes in oat, the *EPF/EPFL* gene family has been confirmed in multiple plant species to regulate plant development and growth, especially in morphogenesis processes such as stomata, awns, stamens, and fruit skin formation [27–30]. In this study, we analyzed the tissue-specific expression patterns of 33 *AsEPF/EPFL* genes in oat and found significant tissue specificity. Most genes were highly expressed in roots and spikes: 12 genes showed the highest expression in roots, while 8 genes exhibited elevated expression in leaves. This expression pattern suggests that *AsEPF/EPFL* genes may be involved in regulating the development of roots, leaves, and spikes in oat. For example, *CmEPF1* is predominantly expressed in melon roots, where it modulates root cortical aerenchyma to regulate growth [31]. After overexpressing *AtEPFL9* in *Arabidopsis*, the overexpressing lines exhibited a higher number of stomata in mature leaves, indicating that *AtEPFL9* positively regulates stomatal development in plants [32]. Similarly, overexpression of *OsEPF1* reduces the stomatal density and enhances root aerenchyma formation, impacting early leaf sheath development in rice [33], whereas *MdEPF2* overexpression in apple significantly decreases the stomatal density [34].In ryegrass, *ScEPF1* and *ScEPF2* overexpression reduce the stomatal density and improve drought tolerance [35]. Additionally, Lu et al. reported that *OsEPFL2* was highly expressed in rice panicles, and CRISPR/Cas9-mediated knockout of *OsEPFL2* resulted in transgenic rice with short or no awns [18]. In this study, we detected high expression levels of multiple *AsEPF/EPFL* genes in oat roots and spikes, suggesting an evolutionarily conserved role of this gene family in root and panicle development within Poaceae plants. Future studies employing CRISPR/Cas9-mediated knockout experiments could further elucidate the molecular mechanisms by which these genes regulate panicle morphogenesis and stomatal development in oats.

With the growing global population and extreme climate events, crop production and food security face significant challenges. Plants must adapt to harsh conditions such as drought and soil salinity, which present significant challenges to crop growth and threaten global food security [36]. Salt stress is a major abiotic factor limiting crop productivity, with plant-specific *EPF/EPFL* peptide hormone families, playing crucial roles in stress adaptations through their regulation on stomatal development and abiotic stress signaling pathways.In this study, by integrating the transcriptomic data of monocotyledonous plants under salt stress from the GEO database (GSE21651), we found that *Os08g0485500* in the salt-tolerant rice variety and its homologous genes in oat (*3AG0421080*, *3CG0493720* and 3DG0522310) were significantly upregulated in a synchronized manner after 24 h of NaCl treatment, indicating conserved role of the *EPF/EPFL* family in salt stress adaptation.

Furthermore, we found that the high expression of *2CG0320600* and *2AG0242220* under drought stress may be associated with the enhancement of plant stress tolerance.The oat genes *2CG0320600* and *2AG0242220* were phylogenetically clustered into the same evolutionary subgroup as *AtEPF1* and *AtEPF2* in *A. thaliana*. This evolutionary congruence implies functional conservation, particularly in stomatal density regulation, with oat *2CG0320600* and *2AG0242220* likely performing analogous roles to *Arabidopsis AtEPF1/AtEPF2* [37]. In *Arabidopsis*, *AtEPF1* and *AtEPF2* encode canonical regulators of stomatal patterning. Mechanistically, these EPF family peptides interact with the receptor kinase *TMM* (TOO MANY MOUTHS) and *ERECTA* family receptors to suppress stomatal lineage initiation [38]. For example, *AtEPF2* overexpression reduces the stomatal density, whereas *atepf2* mutants present hyperstomatal phenotypes. The evidence suggests that these genes modulate the stomatal density to mitigate the effects of drought and salinity conditions. By extension, oat *2CG0320600* and *2AG0242220* may orchestrate similar adaptive mechanisms, enhancing stress tolerance through conserved stomatal developmental pathways.Future studies employing overexpression experiments could further elucidate the molecular mechanisms through which these genes regulate stomatal development in oats.

## Conclusions

This study identified 33 *AsEPF/EPFL* genes from the oat genome using bioinformatics data, analysed their gene structures and conserved protein domains, and investigated the evolutionary relationships of *EPF/EPFL* genes across species. An analysis of the expression patterns of *AsEPF/EPFL* genes in different tissues (roots, stems, leaves, and spikes) suggested that *AsEPF/EPFL* genes play important roles in root, leaf, and spike morphogenesis in oats. Additionally, the expression characteristics of 33 *AsEPF/EPFL* genes under drought and salt stress were analysed, revealing the significant induction of *AsEPF/EPFL* expression with a prolonged stress duration. Based on the conserved functions of homologous genes in other species, candidate genes potentially involved in the oat drought stress response were identified. This research lays a foundation for further elucidation of the biological functions of the oat *EPF/EPFL* gene family.

## Data Availability

All data generated or analyzed during this study are included in this published article and its supplementary information files Declarations.
